# New Features of Laboratory-Generated EPFRs from 1,2-Dichlorobenzene (DCB) and 2-Monochlorophenol (MCP)

**DOI:** 10.1021/acsomega.3c08271

**Published:** 2024-02-13

**Authors:** Lavrent Khachatryan, Marwan Y. Rezk, Divine Nde, Farhana Hasan, Slawomir Lomnicki, Dorin Boldor, Robert Cook, Phillip Sprunger, Randall Hall, Stephania Cormier

**Affiliations:** † Department of Chemistry, Louisiana State University, Baton Rouge, Louisiana 70803, United States; ‡ Department of Engineering Science, Biological Engineering, Louisiana State University, Baton Rouge, Louisiana 70803, United States; § Department of Environmental Sciences, Louisiana State University, Baton Rouge, Louisiana 70803, United States; ∥ Department of Physics and Astronomy, Louisiana State University, Baton Rouge, Louisiana 70803, United States; ⊥ Natural Sciences and Mathematics, School of Health and Natural Sciences, Dominican University of California, San Rafael, California 94901, United States; # Department of Biological Sciences, LSU Superfund Research Program and Pennington Biomedical Research Center, Baton Rouge, Louisiana 70808, United States

## Abstract

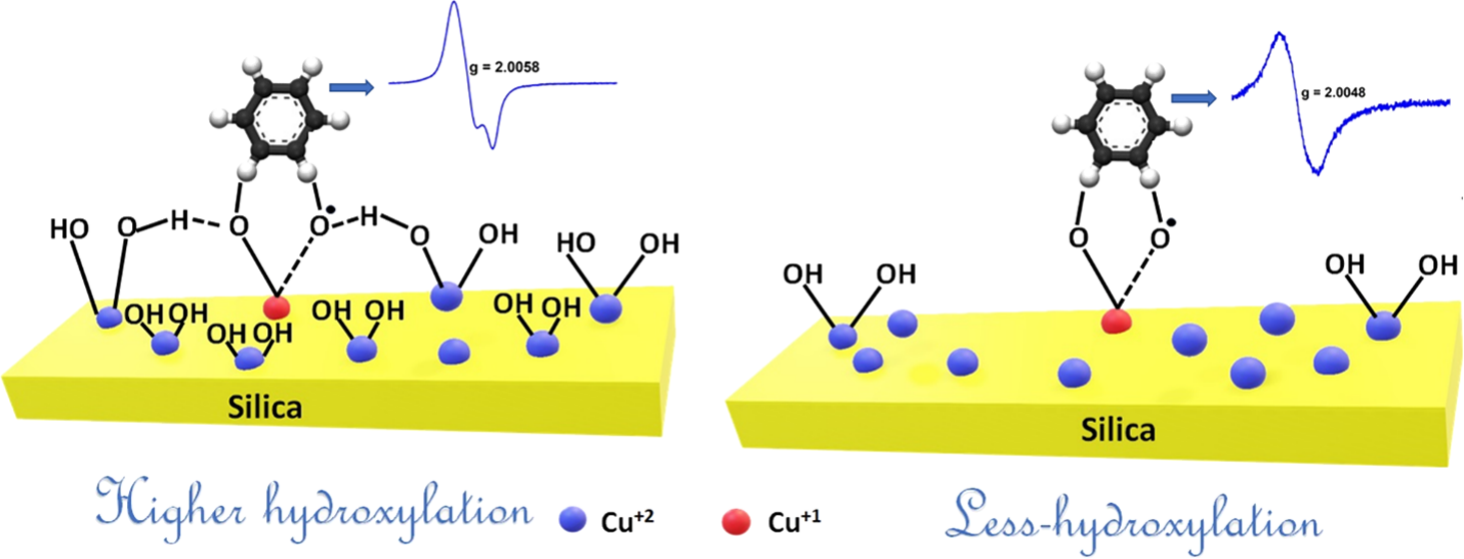

The present research is primarily focused on investigating the characteristics of environmentally persistent free radicals (EPFRs) generated from commonly recognized aromatic precursors, namely, 1,2-dichlorobenzene (DCB) and 2-monochlorophenol (MCP), within controlled laboratory conditions at a temperature of 230 °C, termed as DCB230 and MCP230 EPFRs, respectively. An intriguing observation has emerged during the creation of EPFRs from MCP and DCB utilizing a catalyst 5% CuO/SiO_2_, which was prepared through various methods. A previously proposed mechanism, advanced by Dellinger and colleagues (a conventional model), postulated a positive correlation between the degree of hydroxylation on the catalyst’s surface (higher hydroxylated, HH and less hydroxylated, LH) and the anticipated EPFR yields. In the present study, this correlation was specifically confirmed for the DCB precursor. Particularly, it was observed that increasing the degree of hydroxylation at the catalyst’s surface resulted in a greater yield of EPFRs for DCB230. The unexpected finding was the indifferent behavior of MCP230 EPFRs to the surface morphology of the catalyst, i.e., no matter whether copper oxide nanoparticles are distributed densely, sparsely, or completely agglomerated. The yields of MCP230 EPFRs remained consistent regardless of the catalyst type or preparation protocol. Although current experimental results confirm the early model for the generation of DCB EPFRs (i.e., the higher the hydroxylation is, the higher the yield of EPFRs), it is of utmost importance to closely explore the heterogeneous alternative mechanism(s) responsible for generating MCP230 EPFRs, which may run parallel to the conventional model. In this study, detailed spectral analysis was conducted using the EPR technique to examine the nature of DCB230 EPFRs and the aging phenomenon of DCB230 EPFRs while they exist as surface-bound *o*-semiquinone radicals (*o*-SQ) on copper sites. Various aspects concerning bound radicals were explored, including the hydrogen-bonding tendencies of *o*-semiquinone (*o*-SQ) radicals, the potential reversibility of hydroxylation processes occurring on the catalyst’s surface, and the analysis of selected EPR spectra using EasySpin MATLAB. Furthermore, alternative routes for EPFR generation were thoroughly discussed and compared with the conventional model.

## 1 Introduction

The formation and toxicological consequences of resonantly stabilized persistent free radicals, PFRs^1−3^ (abbreviated later as environmentally persistent free radicals, EPFRs^4^), are strongly correlated to be a significant contributor to the overall potency of particulate matter (PM). It is now a well-known fact that EPFRs are derived primarily from the incomplete combustion of organic materials; they are typically formed on particulate matter through interaction with aromatic hydrocarbons, catalyzed by transition metal oxides, and produce reactive oxygen species (ROS) in biological media that may initiate oxidative stress.

A large distribution of EPFRs in different environmental samples such as environmental particulates PM_2.5_, contaminated soil and sediment samples, Superfund soil samples in the USA, samples from plants’ phytometric measurements, EPFRs on engineered nanoparticles, biomass burning residues, biochars, carbonaceous adsorbents, etc. has been identified. A comprehensive description of the formation, characteristics, decay phenomenon, and applications of surface-bound EPFRs is continuously presented in several publications.^4−11^

**Figure 1:**
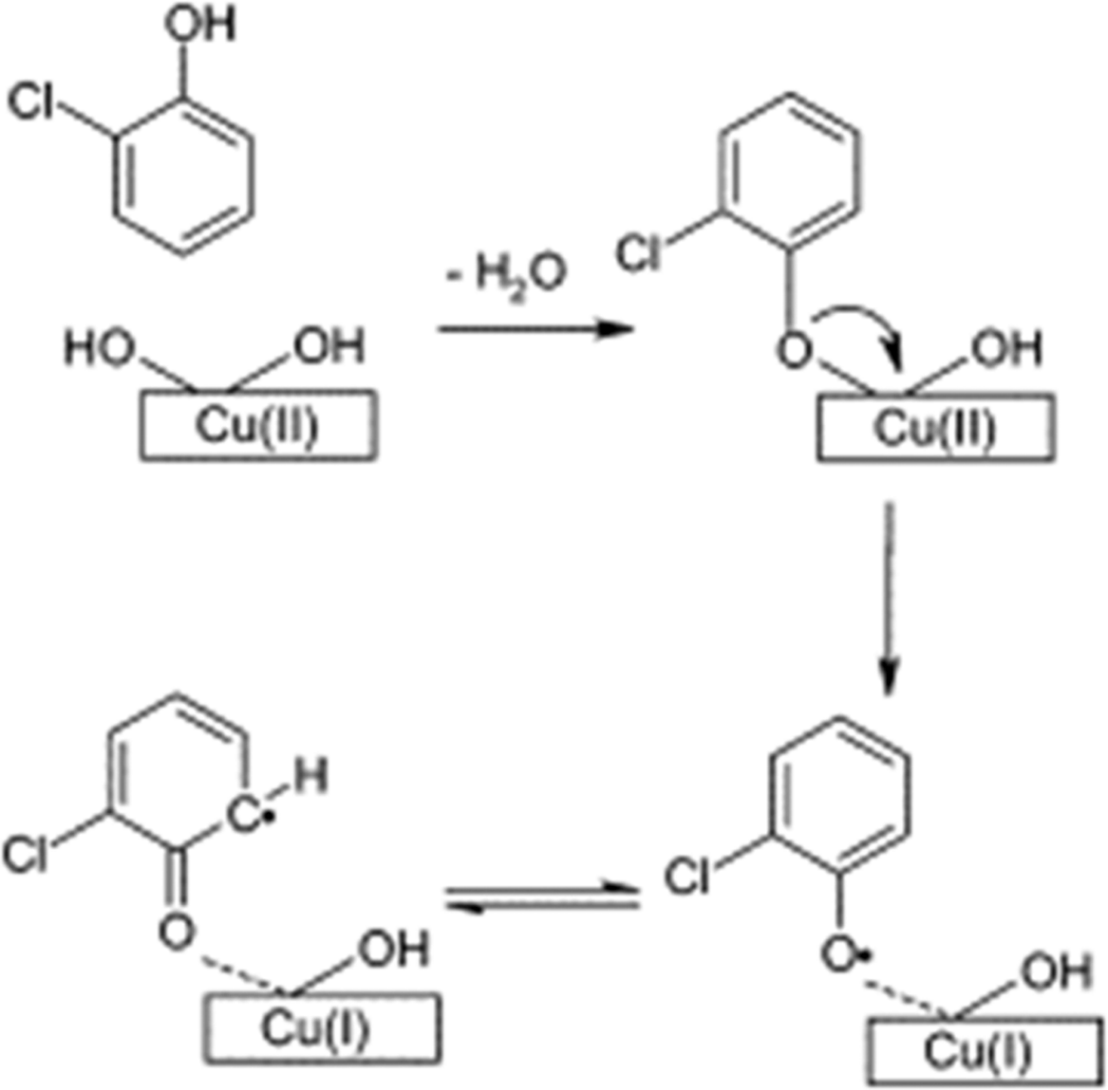
General mechanism (conventional model) of the formation of persistent free radicals, PFRs (lately known as environmentally persistent free radicals^4^), on a copper(II) oxide/silica surface from the adsorption of 2-chlorophenol. Similar interactions are suggested for DCB by the elimination of HCl in the first stage.

An early mechanism proposed by Dellinger et al.^1−4^ suggested that molecular aromatic adsorbate chemosorbs first on the oxide surface supported by a silica matrix; this chemisorption process accompanied by a one-electron transfer from the aromatic molecule to the transition metal center occurs by concomitant reduction of the metal and EPFR formation, Figure 1. The reduction of metals has been confirmed experimentally as well as in several theoretical publications.^12−15^ For instance, X-ray spectroscopic studies (XANES - X-ray absorption near edge structure) of the high-temperature reduction of Cu(II)O by 2-chlorophenol on silica (as a simulated fly ash surface) have shown a reduction of Cu(II) to Cu(I) and Cu(0) with the dominant species being Cu(I).^12^ Further experimental confirmation on the reduction of transient metals, particularly Cu(II), was continued in one of the early works of Dellinger and colleagues by studying the reaction of 2-chlorophenol (MCP), 1,2-dichlorobenzene (DCB), and monochlorobenzene on CuO/SiO_2_ surfaces.^16^ Using XANES spectroscopy, it was shown that chemisorption of MCP and DCB resulted in the formation of identical surface-bound species, chlorophenolates.

The mechanism of chemisorption (Figure 1) involves the formation of chlorophenolate first, followed by the formation of intermediate stabilized radicals, EPFRs. In the absence of CuO, DCB does not undergo chemisorption onto silica. This implies that the initial adsorption of DCB by the analogy of MCP takes place at the copper site, Figure 1, while possible adsorption of DCB on the other sites of CuO as oxygen vacancies was not excluded.^16^ It was also shown that the rate of chemisorption of DCB is slower than that of MCP by a factor of 10.

Due to the large difference in absorptivity of these two compounds,^4^ the current research was initiated to closely investigate the role of the hydroxylation degree of CuO in the generation of EPFRs from DCB (abbreviated DCB230 EPFRs) by comparison with the EPFRs from MCP (abbreviated MCP230 EPFRs). Various protocols were employed to prepare distinct batches of catalyst 5% CuO/SiO_2_, resulting in surfaces with varying degrees of hydroxylation. These were designated as higher hydroxylated batches (HHB) and less hydroxylated batches (LHB) to fulfill specific objectives.

Therefore, when the catalyst surface exhibits a higher level of hydroxylation, we can anticipate increased yields of EPFRs, particularly in the context of generating DCB230 EPFRs. Concomitantly, the alternative pathways for the generation of EPFRs are discussed and compared to the conventional model shown in Figure 1.

## 2 Experimental Section

### 2.1 Materials

**Table 1:** Catalyst Preparation Protocols

				calcination				
batches^a^	added water, mL	mixing mode	drying, °C	°C	time	catalyst color	exposed aromatic	EPFRs concentration, ×10^17^ spins/g
HHB	12	occasionally	120	450	6 h	green	DCB	3.38 ± 0.56
LHB	12	occasionally	120	450	4 days	gray	DCB	0.32 ± 0.10
HHB; LHB							MCP	1.37 ± 0.40

a HHB, Higher hydroxylated batch—colored in green and LHB, less hydroxylated batch—colored in gray.

### 2.2 Copper Oxide/Silica Synthesis, Table 1

Particles of 5% CuO/SiO_2_ (thereafter, catalyst) were prepared by impregnation of 0.1 M copper nitrate hemipentahydrate with silica powder. The obtained gel was dried at 120 °C overnight, calcinated at 450 °C for 6 h, referred to as a standard catalyst (and longer depending on a protocol) in air, and then ground and sieved (mesh size 230, 63 μm). This is a well-known standard method for the preparation of CuO/SiO_2_ particulates reported in early publications.^1,3^ The standard synthesis technique was slightly modified by adding extra water (Table 1), and the final product was calcinated for 6 h (referred to as higher hydroxylated batch (HHB), Figure S1a) and for 4 days (referred to as less hydroxylated batch (LHB), Figure S1b). Furthermore, the catalysts were visually distinguished by their colors: HHB was represented in green, while LHB was represented in gray (Figure S1). The hydroxylation degree of the catalysts was considered as HHB > LHB, which corresponded to their respective degrees of hydroxylation in descending order, vide infra.

To provide a better qualitative difference in colors, a UV–vis spectrophotometer (Flame UV–vis miniature spectrophotometer, Ocean Insight) in reflectance mode was used to verify the wavelength of the observed color. Reflectance was measured at the visible range of 300–700 nm perpendicular to the catalyst samples using an Ocean Optics spectrophotometer with a bifurcated probe and deuterium-halogen light source. White Spectralon (a fluoropolymer that has an extremely high diffuse reflectance) and opaque black were used as references to 100 and 0% reflectance, respectively.

In addition, the powder samples were further characterized with the following techniques: TEM, transmission electron microscope; XRD, X-ray diffraction spectroscopy; TGA, thermogravimetric analysis; XPS, X-ray photoelectron spectroscopy; and EPR, electron paramagnetic resonance radio spectroscopy.

### 2.3 EPFR Preparation

Normally, the obtained 5% CuO/SiO_2_ powder was dried at 120 °C in the exposure chamber^3^ and then reheated at 450 °C for 30 min in a vacuum to remove any remaining organics. Ultimately, the copper oxide/silica catalyst underwent several cycles of exposure to saturated vapors of DCB and MCP at 230 °C under a vacuum of 10^–2^ Torr, each lasting 5 min. This process ensured surface saturation as the stabilization of the EPR signal intensity was observed after 5 repetition cycles. Then, the exposure chamber was allowed to cool down at 50 °C under vacuum, and the particulate was subjected to EPR analysis (X band EPR, Bruker EMX-20/2.7).

## 3 Results and Discussion

### 3.1 Morphological and Structural Analysis of Modified 5% CuO/SiO_2_ Matrixes

A comprehensive analysis of catalyst morphology when exposed to aromatic compounds, specifically focusing on the presence of the reduced Cu^1+^ state during EPFR generation and other detailed information, has been previously presented in earlier publications.^12,17^ In the Supporting Information, TEM images illustrating the distinctions between HHB and LHB samples, as well as the reduction of Cu^2+^ to Cu^1+^ as determined by XRD measurements, are provided (refer to Figures S2 and S3). It is important to highlight that numerous publications have successfully elucidated the reduction process of transition metals in the generation of EPFRs from various precursors using different techniques.^12,16−21^ Nevertheless, it is worth noting that during the EPFR generation process, detecting the reduced state of Cu^1+^ can be challenging, primarily due to the rapid oxidation of Cu^1+^ into Cu^2+^ in the presence of trace amounts of air (vide infra, Figure 6 and Supporting Information; section “A reversible color change of the catalyst”).

#### 3.1.1 Thermogravimetric Analysis

**Figure 2:**
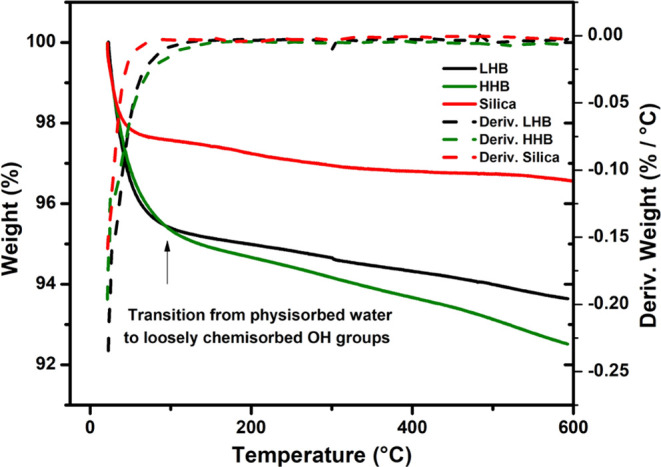
Solid lines: thermogravimetric analysis for the HHB sample (green), LHB sample (black), and silica (red). Dashed lines are the derivative curves of the corresponding batches.

As a reliable, fast, and simple technique, thermogravimetric analysis was performed to reveal the degree of hydroxylation of the surface. The analysis was executed from room temperature up to 600 °C for HHB, LHB, and silica as a reference after the system was purged with nitrogen. The TGA spectrum shown in Figure 2 shows a sharp weight loss while heating all samples from room temperature up to slightly more than 100 °C due to the loss of physisorbed water on the surface of the catalysts.

At 160 °C, the weight loss percentages of HHB and LHB were calculated using TA Universal software to be 5.2 and 4.8%, respectively. In the temperature range from 120 to 600 °C, HHB (green line) has a higher weight loss. This suggests that it has more original surface OH groups than LHB.^22,23^ The weight losses between 120 and 600 °C can be ascribed to either loosely or strongly bound OH groups.^22,23^ A similar source for the surface OH groups can be adsorbed water molecules on the catalyst surface, which are split on the surface to form hydroxyl groups on Cu^2+^ sites and hydrogen atoms on surface oxygen—this scenario was considered in ref (24) during molecular adsorption of phenol on γ-Al_2_O_3_.

The role of hydroxyl groups, specifically in catalysis, has been of crucial interest in the literature. Depending on the application, hydroxyl groups proved to enhance the catalytic process either by forming a more stable catalyst^25^ or by inducing oxygen vacancies to improve the catalytic properties of metal oxide.^26^ Ullattil and Periyat^27^ reported that the work done in sol–gel hydroxylation produced a rich oxygen vacancy-rich black anatase TiO_2_, which interestingly was obtained in the absence of dopants, reducing agents, and with no need for any high pressure or temperature. In addition, the controlled presence of loosely bound oxygen in the form of OH proved to enhance the thermal stability of the nanostructured metal oxide catalysts. The early findings presented by one of the coauthors of this study, Rezk et al.,^28^ substantiate this assertion; the loosely bound OH groups result in reduced weight loss, as observed in TGA measurements (indicative of water removal). In other words, the loosely bound OH groups likely create additional space for dangling bonds to engage with the polymer, thereby enhancing matrix stabilization.

#### 3.1.2 UV–Vis Reflectance Detection

**Figure 3:**
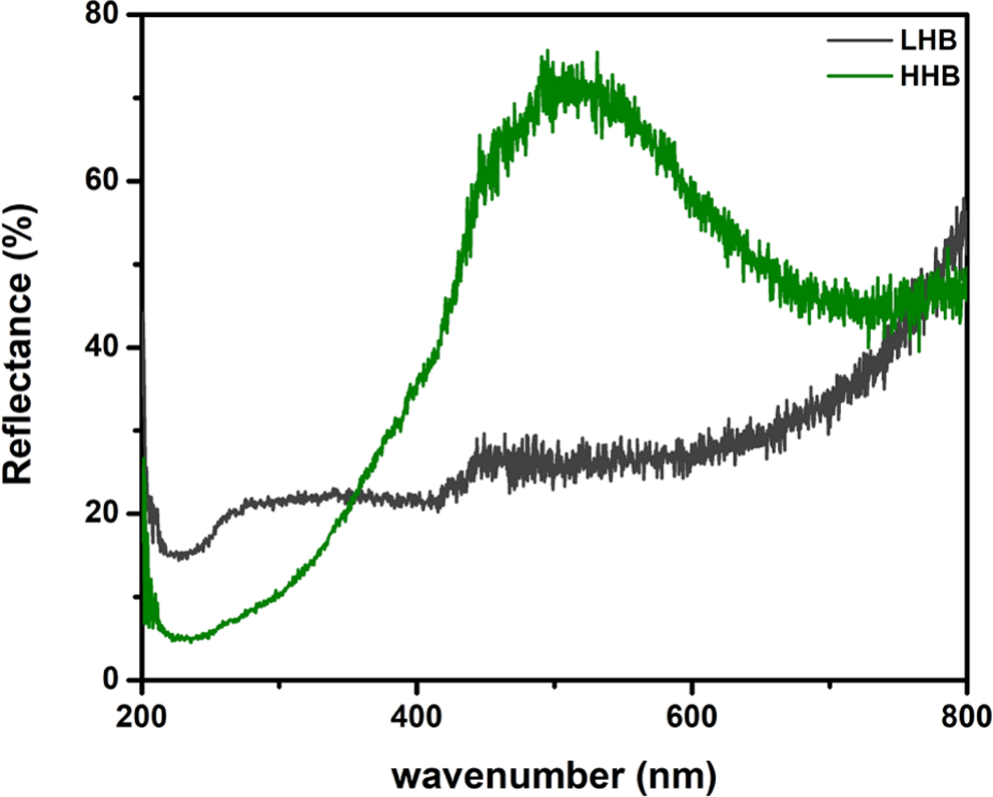
UV–vis reflectance spectra of HH (green) and LH catalysts. HH and LH stand for higher and less hydroxylated surfaces of the catalyst, respectively.

An empirical UV–vis reflectance measurement of the surface condition due to the intrinsic nature of the surface itself^29^ for both batches (HHB and LHB) has been employed. The difference between the surfaces of HHB and LHB can be seen from their UV–vis reflectance spectra (referred to as albedo^29^), Figure 3. HHB (green in color) shows an excitation range with a broad peak between 450 and 580 nm, while LHB (gray color) has a broad reflectance spectrum but not obvious reflectance that covers almost the whole range from 200–800 nm width. Although specific causes for color interpretation are complex, some characteristics for the formation of color can be deduced. For instance, HHB, which has a green color, probably contains more water molecules than LHB by the analogy of a zeolite type of catalyst colored green, which contains more water molecules attached to Cu^2+^ as ligands by changing the color to pale green.^30^ It is also known that the cations of the majority of transition metals as d-block elements predominantly contain unpaired electrons and are colored by bonding the water molecules as ligands. This suggests that the partly filled d-orbitals must be involved in generating the color. When the ligands bond with the transition metal ion, there is repulsion between the electrons in the ligands and the electrons in the d-orbitals of the metal ion. This raises the energy of the d-orbitals and splits them (removal of the “degeneration”)^31^ into two groups, the highest occupied molecular orbital (HOMO) and lowest unoccupied molecular orbital (LUMO). From crystal field formalism, the less the splitting, the less energy (longer wavelength, the red region of the spectrum) needed to excite an electron from the LUMO to the HOMO. This phenomenon happens with HHB—the LUMO state absorbs the red spectrum and the complementary to the red color cyan (bluish green) is emitted^31,32^ and registered by the spectrometer, Figure 3. The size of the energy gap between HOMO/LUMO varies with the nature of the transition metal ion, its oxidation state, and the nature of the ligands.

In the case of LHB, due to much less water content, the Cu^2+^ ions bond less with water molecules, and hence, no perturbation of the d-orbital occurs. As no obvious reflectance has been detected (Figure 3), the trend is typical for the same phenomenon from different surfaces (snow, soil, etc.).^33^

### 3.2 Nature of DCB230 EPFRs

**Figure 4:**
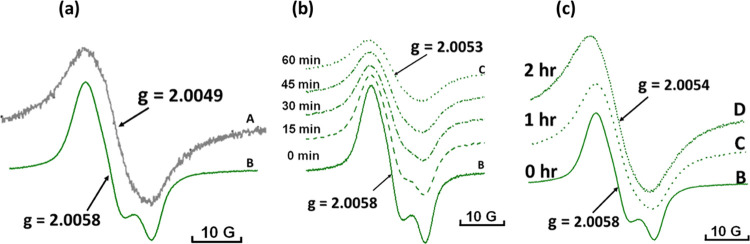
(a) EPR spectra of DCB230 EPFRs generated from the catalysts prepared by using different protocols: spectrum **A**—the LHB catalyst colored in gray after long drying, spectrum **B**—the HHB catalyst colored in green after short drying. (b) The spectra obtained after one h of aging of spectrum B (labeled as C) and two h of aging (spectrum D) exhibit remarkable similarity. (c) The first-hour detail aging of EPR spectra of DCB230 EPFRs generated from HHB (colored in green).

The EPR spectra of the EPFRs generated from two different catalysts (prepared by different protocols) are represented in Figure 4a. A smooth conversion of nonstructured EPR line **A** (*g* = 2.0049, Δ*H*_p–p_ = 14.5 G) to the EPR line **B** with a fine splitting at a high magnetic field (*g* = 2.0058, Δ*H*_p–p_ = 16.0 G) has been observed using HHB. During a 5-month observation period, (A) and (B) types of DCB230 EPFRs with elevated g values, specifically ranging from 2.0049 to 2.0053 (gray-colored cycles) and 2.0053 to 2.0058 (green-colored cycles), respectively, were periodically detected, Figure S4.

The complex characteristics of the initial radical **B** are clearly apparent from the aging experiments depicted in Figure 4b for the first-hour measurements; the spectral splitting observed in spectrum **B** gradually diminishes after one hour of aging, spectrum **C**. It is noteworthy that a gradual transition from split spectrum B to A has also been observed by comparison of results from HHB and LHB surfaces with an intermediate hydroxylated catalyst prepared through the standard synthesis protocol^1,3^ (details not shown). A further second-hour aging does not alter spectrum **C**, as illustrated in Figure 4c, spectrum **D**. It is worth noting that our earlier publication provided a preliminary identification of the potential radicals present in a mixture of DCB230 EPFRs.^3^ However, our current research is focused more closely on elucidating the nature of these radicals in complex EPR spectrum **B**, Figure 4a.

#### 3.2.1 Fresh vs Aged DCB230 EPFRs

**Figure 5:**
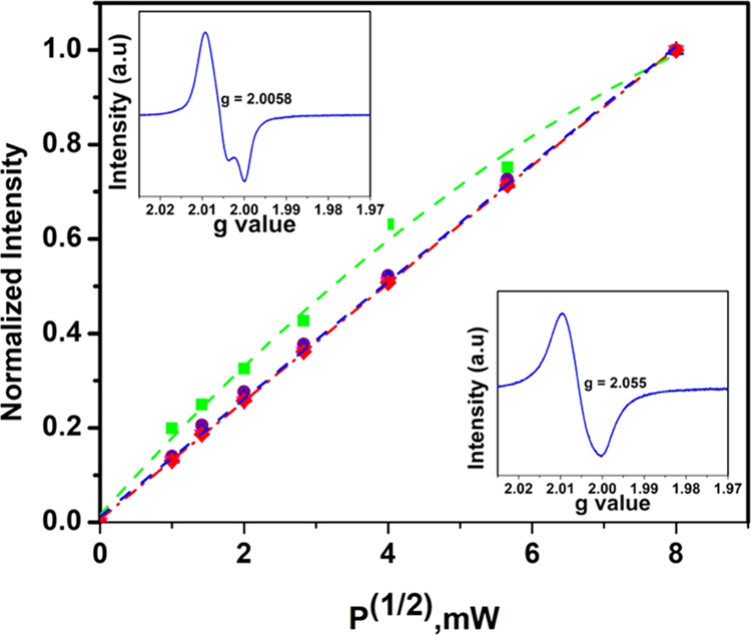
Power dependence of fresh EPFRs (EPR spectrum, upper inset) generated from DCB, green line, and aged EPFRs (EPR spectrum, below inset) after 1 and 2 h aging, violet dashed and red dashed lines, respectively. HHB was used for EPFR× generation.

To validate the identity of the EPR spectra of fresh (**B**) and aged EPFRs (**C** and **D**), Figure 4c, an EPR microwave power dependence of the intensity of the radicals was performed, Figure 5. Free radicals are often characterized by long spin–lattice relaxation times, which can produce a “saturation broadening” vs microwave power because the spins in the excited level cannot return to the ground level sufficiently quickly. This effect will depend on the complexity of radicals and will be sensitive to microwave energy. Different “saturation” curves, i.e., EPR spectral intensity dependence vs *P*^1/2^ (*P*-microwave power in milliwatts), will be expected in the case of a mixture of radicals^34,35^ or different paramagnetic centers.^36^

DCB230 EPFR samples may also have different saturation dependencies versus microwave power. For example, the power dependence of fresh EPFRs (the EPR spectrum in the upper inset) generated from DCB (green line) and linear dependence of aged EPFRs (the EPR spectrum in the below inset) after 1- and 2 h aging is depicted in Figure 5. The small difference in the behavior between fresh and aged radicals, Figure 5, is obvious by comparison of the spectral shape of fresh EPFRs with a split (upper inset) and an aged radical (without a split, below inset, Figure 5). It is also remarkable that the same power dependence of radicals aged for one and two h, Figure 5 (violet and red lines), was observed. This fact additionally proves the identity of radicals **C** and **D**, Figure 4c. Additionally, from power dependence measurements, it can be concluded that there is not a large difference between the behavior of fresh and aged radicals toward microwave power, Figure 5.

#### 3.2.2 Mechanism of Formation of DCB230 EPFRs. H-Bonding

**Figure 6:**
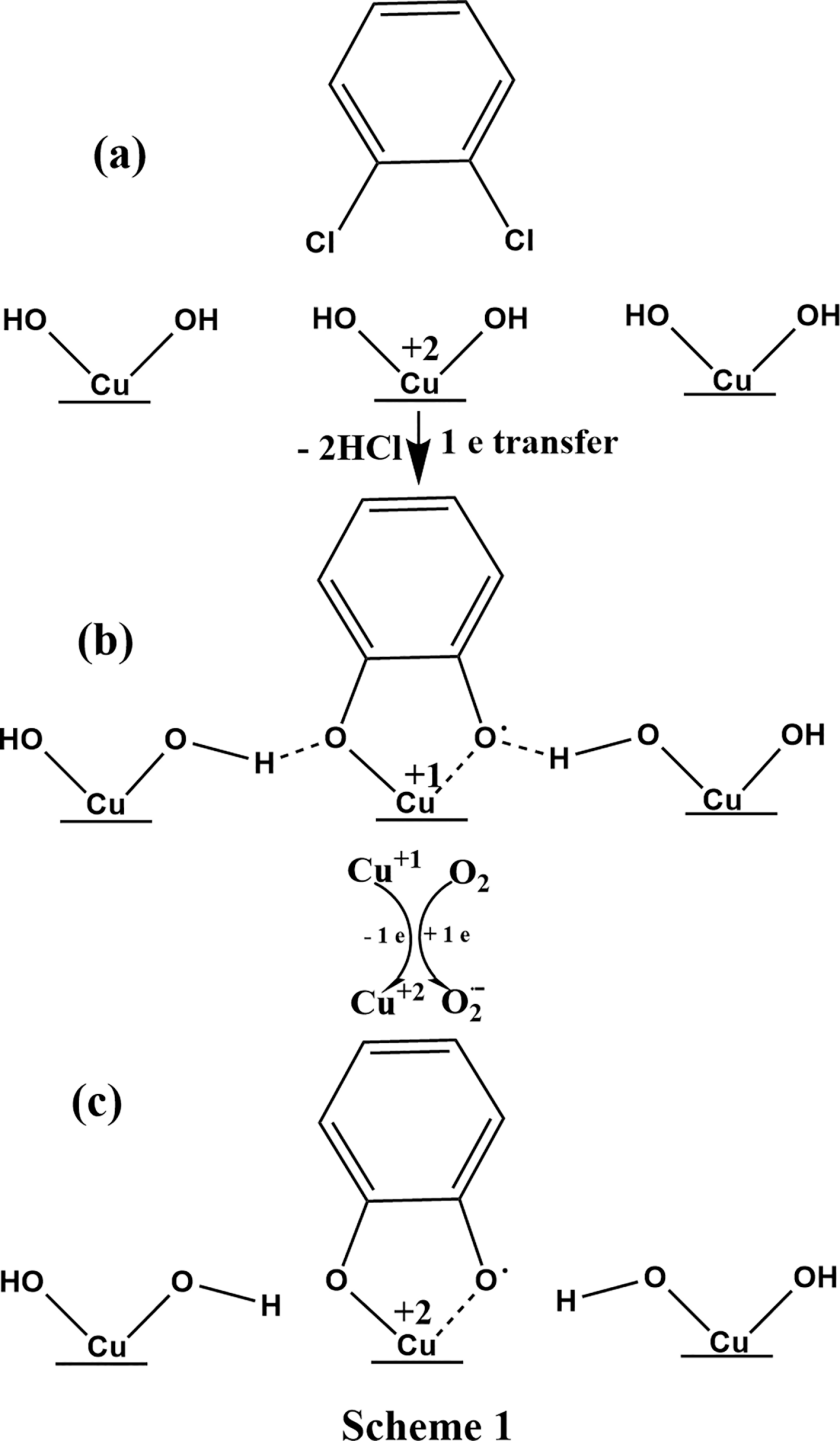
(a) Bidentate chemisorption of 1,2-dichlorobenzene according to the conventional model. (b) Hydrogen bonding. (c) Breaking of H-bonding by air (ref Section 3.2.3) and concomitant oxidation of Cu^1+^ and formation of adsorbed superoxide radical, O_2_^**–**^.

The experimental findings align with the established model for generating DCB EPFRs,^1,3,4^ in which increased hydroxylation is expected to lead to increased EPFR yields. Indeed, enhancing the degree of hydroxylation in the catalyst, progressing from LHB to HHB, resulted in a significant rise in the average yields of DCB230 EPFRs (Table 1), increasing from (0.32 ± 0.1) × 10^17^ spins/g (LHB sample) to (3.38 ± 0.56) × 10^17^ spins/g (HHB sample). Note that DCB230 EPFRs generated from HH surfaces exhibit a split in their EPR spectra, whereas those formed from LH surfaces display EPR spectra without a split, as shown in Figure 4a. In our previous study, a smooth transition of a split EPR spectrum with a high g value to a nonsplit EPR spectrum with a relatively low g value was observed.^37^ This transition occurred depending on the weight percentage content of CuO in the catalyst used. This qualitative change in radicals formed was addressed to the transfer from semiquinone (high g value) to chlorophenoxy radicals (low g value), presented schematically in Figure S5. By similarity to this scenario, it could be hypothesized that at the highest hydroxylation of the surface, the bidentate adsorption of DCB on geminal hydroxyl sites by the formation of the *o*-semiquinone type radical (high g values) is much preferable, Scheme 1 (see Figure 6), path (a). Partly, it is because transition metals complexes easily with quinone type of organics and that the semiquinone complexes are intermediates containing paramagnetic ligands.^21,38,39^ Semiquinone free radicals are readily captured by di- or tripositive metal ions in solutions, forming chelate complexes.^40^

On the other hand, H-bonding between surface physisorbed *o*-semiquinone, *o*-SQ(s), and neighbor water molecules (or surface OH groups, Scheme 1, path (b)) is highly possible for the extremely hydroxylated surfaces. With this scenario, the local environment in *o*-SQ(s) may be impacted by hydrogen bonding. For instance, such bonding may manifest as a stabilizing effect, as observed in various enzymes with tyrosyl radicals.^41,42^ This type of intermolecular hydrogen bonding results in a splitting of the spectrum due to hyperfine spin coupling between unpaired electrons on terminal oxygen of *o*-SQ(s) and hydroxyl hydrogen of either the absorbed water molecule or the surface OH group, Scheme 1, path (b). A typical observation (hydrogen isotropic hyperfine splitting), however, in the liquid phase, has been detected between phenoxyl radicals and their parent phenols, usually present in large amounts in solution as radical precursors.^42−44^ Note that the presence of hydrogen bonds between the quinone oxygen and different solvents (such as water and various alcohols) has been experimentally observed using ENDOR spectroscopy at 35 GHz.^45^ These observations have also been confirmed through DFT calculations. It is noteworthy to compare the structural similarity of two EPR spectra of hydrogen bonding of *o*-SQ radicals from catechol vacuum pyrolysis reported from our laboratory^46^ and *o*-SQ(s) derived from exposure of the catalyst to the vapor of DCB and referred to us DCB230 EPFRs, Figure S6. DFT calculation of the g tensor for *o*-SQ has been performed and giso = 2.0051 was reported.^46^

Therefore, it can be concluded that the split EPR spectra B and nonsplit EPR spectra of DCB230 EPFRs, C and D (Figure 4), are practically the same, corresponding to bound *o*-SQ radical. The shift in the g value, from 2.0056(8) in the split spectrum to 2.0052 in the nonsplit singlet spectrum, solely results from the removal of H-bonding.

The experimental results performed below serve as additional evidence of the presence of nonstable H-bonding in the system, which can be disrupted in the presence of N_2_ or O_2_, as previously demonstrated (hydrogen bond breaking by oxygen and nitrogen^47^).

#### 3.2.3 Aging of DCB230 EPFRs

**Figure 7:**
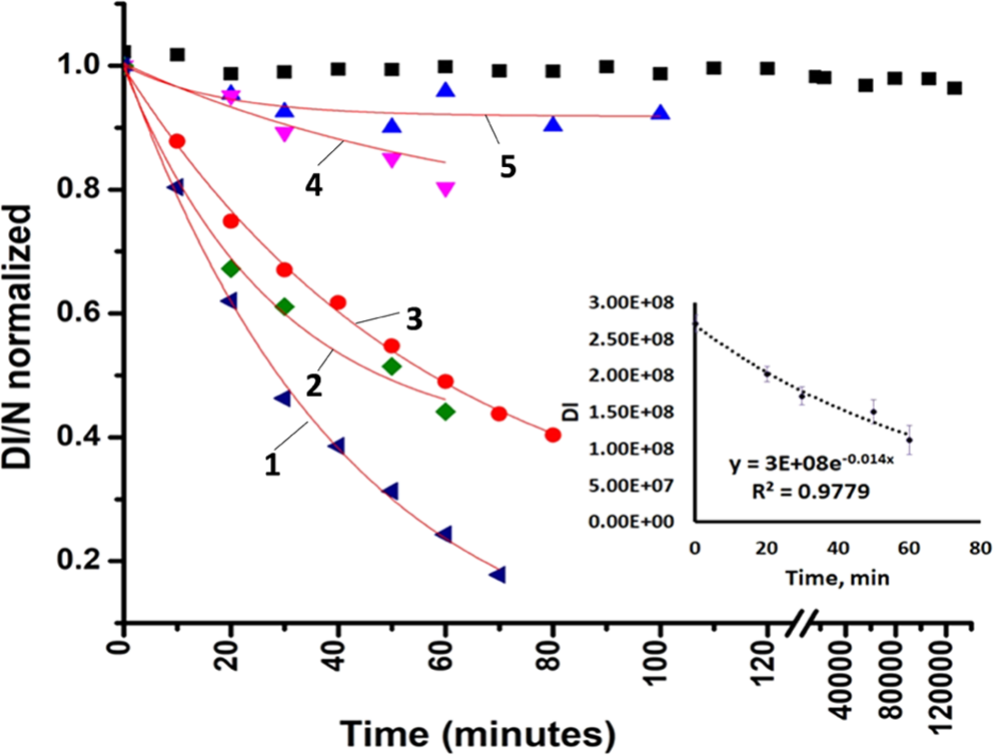
Aging phenomenon of DCB230 EPFRs in an EPR-sealed tube under vacuum (10^–2^ Torr) over a period spanning from 03.14.23 to 06.23.23 (Black rectangles). Observations were also conducted on the EPFRs exponential decay curves using another set of five different EPR tubes equipped with stopcocks (at slow penetration of air into the tube) over a period spanning from 05.12.22 to 02.14.23 (curve 1, lifetime = 30 min; curve 3, lifetime = 62 min; curve 5, lifetime = 115 min). The inset decay curve is an average of these five decay curves. The extracted average 1/*e* lifetime of DCB230 EPFRs is ∼75 min. DI/N for the *Y*-axis stands for the normalized (N) double integration value (DI) of the given EPR spectrum.

The DCB230 EPFRs were kept in the vacuum (10^–2^ Torr) in an EPR tube (o.d. 10 mm, length 10–12 cm) cupped by a regular stopcock, and the intensity of the EPR signal was checked repeatedly in a timely manner. We have detected a remarkable phenomenon of the aging of DCB230 EPFRs depending on the vacuum quality in an EPR tube, as shown in Figure 7. The vacuum experienced disruption, ranging from 10^–2^ to 5–6 Torr the following day, and could even reach levels as high as 50 Torr (confirmed independently). This occurred because of varying air penetration rates, examined by connecting the EPR tube to the vacuum system and monitoring pressure fluctuations in the vacuum line. The ideal condition to avoid penetration of air was the sealing of the EPR tube, which provides aging of DCB230 EPFRs in anaerobic conditions, Figure 7, black scattered rectangles. Practically, no decay of EPFRs was observed for more than 3.5 months, while a different profile of decay was observed in a sealed EPR tube (refer five decay curves for different time spans (Figure 7)). The stability of DCB230 EPFRs is less at a high penetration rate of air (curve 1, 1/*e* lifetime = 30 min) and increases at slow penetration of air (1/*e* lifetime = 115 min, curve 5) and can be indefinitely stable in anaerobic conditions, black rectangles. An average 1/*e* lifetime of DCB230 EPFRs considering an exponential decay of DCB230 EPFRs at slow penetration of air can be extracted from the inset picture, Figure 7 equals ∼75 min.

These findings suggest that the incursion of air into the EPR tube containing the DCB230 EPFR sample likely disrupts the splitting, leading to the spectrum D shown in Figure 4b, possibly due to the disturbance of hydrogen bonding (ref (47)). The continuous aging of DCB230 EPFRs is likely to occur through the process outlined in detail in ref (48).

##### 3.2.3.1 Spectrum Simulation

Nonetheless, at low yields of DCB230 EPFRs and at the end of aging when the residual radical intensity is much lower, the radical EPR spectra are structureless, broad EPR lines with Δ*H* ∼ 15(16) G and g values less than 2.0052 (namely, *g* = 2.0049, Figure 4a). However, there is uncertainty in the measurement of the g value (2.0049) at low yields of DCB230 EPFRs because of the broad, nonresolved EPR spectrum of Cu^2+^ overlapping with the EPR spectrum of the organic radical, as shown in Figure S7a.

Under these circumstances, simulating the EPR spectrum can aid in the accurate extraction of information about the organic radical. The EasySpin computational package^49^ was employed to simulate and analyze the complex EPR spectra, such as that shown in Figure S7a. Because of significant magnetic field drift, the simulation was focused on a portion of the spectrum. The EasySpin simulation of a largely broadened Cu^2+^ EPR spectrum^50^ at low symmetry^30,51^ and axial g tensors (*g*_*x*_ = *g*_*y*_ = 2.0882, *g*_*z*_ = 2.390)^30^ overlaid on an organic signal (here DCB230 EPFRs) is represented in Figure S7b (red line is the simulated spectrum). The EPR experimental signal for Cu^2+^ lacks hyperfine splitting due to the high content of copper in the catalyst (3.5 wt %) and a close vicinity of Cu^2+^ to each other, which leads to large broadening due to dipole–dipole interaction.^50^ The high g value of 2.0052 extracted from simulation to fit experimental data (Figure S7a) additionally validates the fact that even at low yields of DCB230 EPFRs, the preferable surface radicals resemble *o*-SQ radicals. The specifics of the EasySpin code can be found in the Supporting Information.

**Figure 8:**
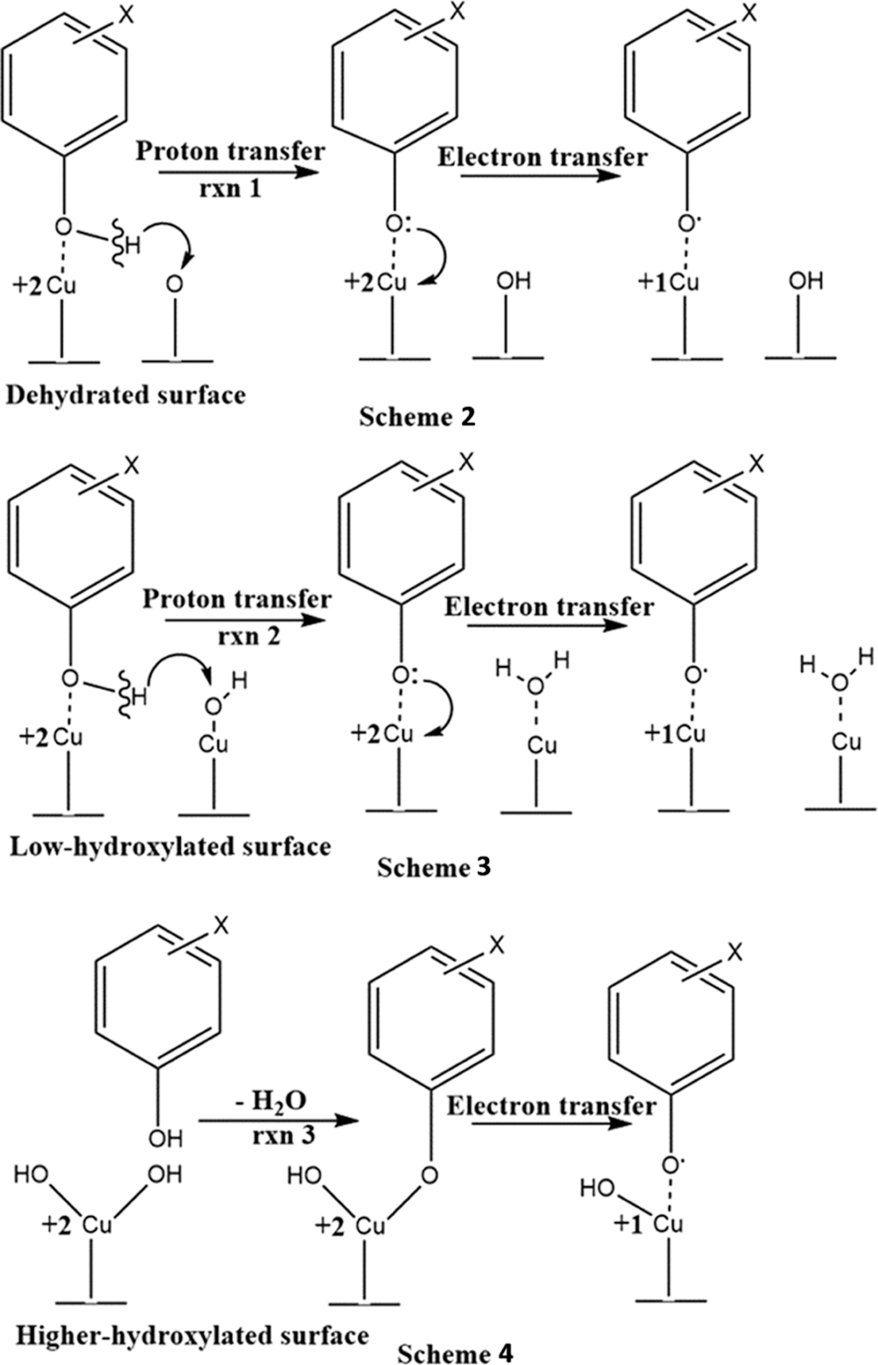
Schemes 2–3 are alternative pathways to conventional Scheme 4.

These results raise the question of whether the experimental spectrum obtained from DCB exposure, which is assigned as bound *o*-SQ radicals, also contains some 1-chlorophenoxy radicals resulting from the monodentate adsorption of DCB (as expected from Figure S5). This scenario may occur on surfaces with fewer hydroxyl groups (LHB) and at a low density of vicinal surface OH groups. On these surfaces, the formation of copper-centered complexes with H-bonding (as shown in Figure 6, Scheme 1, paths (a) and (b)) is less favored, resulting in a nonsplit, singlet EPR spectrum. However, this spectrum is a broad spectrum with Δ*H* of 15G and *g* = 2.0049 (Figure 4a), which is significantly different from the EPR spectrum of MCP230 EPFRs detected by using any type of catalyst (for fresh MCP230 EPFRs *g* = 2.0042, while for aged EPFRs g= 2.0036 at Δ*H* = 5(6) G).

Conversely, we cannot dismiss the potential for 2-chlorophenoxyl radicals to form when the DCB is exposed to the catalyst. These radicals may undergo chlorination readily, as documented by Vejerano.^52^ Depending on the extent of chlorination, this process can lead to a corresponding increase in the g value, as exemplified in refs (53,54) concerning chlorinated phenoxyl radicals in aprotic solutions.

An intriguing discovery was the catalyst’s ability to undergo a reversible color transformation under DCB exposure, manifesting in various shades of green (refer to Figure S8). The color change of the catalyst (HHB), represented by different green hues, was linked to a detailed discussion in the Supporting Information, which centered on the valence alteration of Cu^2+^.

### 3.3 Degree of Hydroxylation of the Catalyst has Varying Effects on the Production of EPFRs from DCB and MCP

Specifically, the increased surface hydroxylation resulted in more than a 10-fold difference in the DCB230 EPFR concentration between the low and high hydroxylation levels. Differences only by a factor of 3 between low and high concentrations of MCP230 EPFRs among the batches (LHB and HHB) used have been seen (Table 1).

Another surprising observation was also the passive behavior of MCP230 EPFRs toward the distribution of nanoislands of CuO over the surface (high or low density), the size of the islands (nanosized or large chunks/layers with different shapes), etc. (ref the TEM images on Figure S2). Independent of the catalyst type (LHB or HHB) and preparation protocol, on average, ∼1.37 × 10^17^ spins/g concentration of MCP230 EPFRs was measured repeatedly, Table 1.

Hence, considering the “indifferent” behavior of MCP230 EPFR generation in relation to catalyst morphologies, we tend to favor the idea that multiple mechanisms, running parallel to the conventional EPFR generation model, are in operation. Several publications have recently raised the importance of numerous alternative pathways for the formation of EPFRs.^5,6,36,55,56,24^ The role of a trace amount of oxygen in the formation of EPFRs was advocated in ref (5) for phenoxy radicals from exposure to metal oxide surface by benzene. A hypothesis was reported recently by Mocarelo^55^ about the critical role of adsorbed superoxide species in the formation of EPFRs. It is also vital to consider the effect of metal surface defects and oxygen vacancies on the formation of EPFRs, which has been shown recently during the process of formation of EPFRs on ZnO surfaces.^36^ The acidity/basicity character of the surface to initiate EPFR generation was reported in ref (56).

The new insight into the reactions occurring on the surfaces of nanosized particles has recently been developed theoretically in ref (57). The phenomenon of “microscopic mechanisms of heterogeneous catalysis” on the surface site of the nanosized particle discovers the fact that the chemical reactions observed at specific active sites might effectively stimulate the same reactions at the neighboring sites (cooperative communication). In this approach, the main role is played by positively charged holes on metal surfaces.

The reactivity of the aromatics toward metal oxide surfaces is theoretically discussed in ref (24) using Periodic DFT calculations. Particularly, different modes by considering the adsorption phenomenon and further transaction of phenol on γ-Al_2_O_3_(110) surfaces are discussed, depending on the hydroxylation degree of the metal oxide surface.

Remarkably, the generation of MCP230 EPFRs on a highly dehydrated surface (like LHB—gray-colored catalyst) can be explained by proton transfer from adsorbed MCP to lattice oxygen and formation of phenolate moieties first^24^ (shown experimentally in early publication^16^) and then stabilized radicals on the metal surface, Scheme 2 (Figure 8).

For less hydroxylated surfaces, the proton may interact with rarely distributed surface OH groups by the formation of surface-bound MCP EPFRs and water molecules, Scheme 3 (Figure 8). As the hydroxylation degree increases (progressing from LHB to HHB), the multimodal scenario^24^ for the generation of EPFRs becomes available parallel to the conventional interaction of the adsorbents with metal-attached OH groups (Scheme 4, Figure 8).

MCP molecules exhibit high adsorption strength^16^ owing to the strong hydrogen bonding or van der Waals interactions between the phenolic hydroxyl groups and the surface oxygen or hydroxyl groups. This leads to the generation of EPFRs through three pathways, as depicted in Schemes 2–4. However, it is unlikely that Schemes 2 and 3 apply to DCB; a transfer of the Cl atom from DCB to the surface oxygen (like in Scheme 2) or to the surface OH group (like Scheme 3) is most probably not favored. On surfaces with high hydroxylation, there is a notable increase in the absorptivity of DCB on hydroxyl sites owing to the elevated surface concentration of OH groups. Consequently, current research demonstrates the formation of DCB230 EPFRs, as illustrated in Scheme 1, Figure 6. Note that the water molecules released in reactions 2 (Scheme 3) and 4 (Scheme 4) may stay adsorbed on CuO, as was shown recently in ref (58) in a high vacuum apparatus under a trace amount of water in the gas phase.

The rate constant calculations for the formation of EPFRs from exposure of phenol to γ -Al_2_O_3_^24^ confirm that Schemes 2 and 3 are dominant over Scheme 4 (water elimination, conventional mode). On the other hand, an additional high level of theoretical microkinetic calculation is needed to understand the large discrepancy of calculated rate constant for Scheme 4.^24^

The modes discussed above for heterogeneous EPFR generation may not be exhaustive, but they are feasible for MCP molecules because of the high mobility of hydroxyl protons, which is not present in DCB molecules lacking such an adsorption group. Consequently, EPFRs from MCP were easily generated across all catalyst types studied, whereas a high yield of DCB230 EPFRs was preferentially produced from hydroxylated surfaces.

## 4 Conclusions

The noticeable variance in absorptivity between well-known aromatic precursors like DCB and MCP on a 5% CuO/SiO_2_ catalyst has prompted the current research to closely investigate the influence of CuO hydroxylation levels on the formation of EPFRs (Environmentally Persistent Free Radicals). This study compared EPFRs generated from DCB, referred to as DCB230 EPFRs, with those generated from MCP, referred to as MCP230 EPFRs. Notably, distinct behaviors were observed during the EPFR generation process by using these two precursors. As the surface’s hydroxylation level increased, the yields of DCB230 EPFRs exhibited a gradual increase, while the yields of MCP230 EPFRs remained unaffected by surface morphological changes. The study suggests that with increasing degrees of hydroxylation, a multimodal scenario for EPFR generation becomes possible for MCP230 EPFRs, in addition to the conventional interaction of adsorbents with metal-attached OH groups.

The study includes a detailed spectral analysis of the nature of DCB230 EPFRs using the EPR technique and an examination of the aging phenomenon of DCB230 EPFRs as surface-bound *o*-semiquinone (*o*-SQ) radicals after bidentate chemisorption of DCB on copper sites. Furthermore, it investigates the H-bonding interactions of *o*-SQ radicals, explores the reversibility of hydroxylation processes on the catalyst’s surface, and employs EasySpin MATLAB analysis to examine the weak EPR spectra. The study also explores alternative pathways for EPFR generation and compares them with the conventional model.

All new hypotheses/approaches discussed in this research improve the mechanistic understanding of EPFR formation on metal oxide surfaces, opening new prospects for further theoretical and experimental studies on the heterogeneous formation of EPFRs in the future.

## Supporting Information Available

HHB (higher hydroxylated batch) and LHB (less hydroxylated batch) samples (Figure S1); TEM images (Figure S2); XRD spectra (Figure S3); EPR spectra of DCB230 EPFRs (Figure S4); general mechanism of EPFR formation (Figure S5); EPR spectra of *o*-SQ radicals under dramatically different conditions (Figure S6); low yield DCB230 EPFRs overlaid on the Cu^2+^ EPR spectrum (Figure S7a); EasySpin simulation (Figure S7b); and reversible color change of the catalyst at the exposure of DCB (Figure S8) (PDF)
