# Supplementary material for: New Features of Laboratory-Generated EPFRs from 1,2-Dichlorobenzene (DCB) and 2-Monochlorophenol (MCP)
Source: ACS Omega. 2024 Feb 13;9(8):9226–35. doi: 10.1021/acsomega.3c08271 (PMC10905596; doi:10.1021/acsomega.3c08271)
Supplement: Supplementary file 1 — ao3c08271_si_001.pdf [file ao3c08271_si_001.pdf]

Supporting Information

New Features of Laboratory Generated EPFRs from 1,2-Dichlorobenzene (DCB) and 2-Monochlorophenol (MCP)

Lavrent Khachatryan[1*], Marwan Y. Rezk [2], Divine Nde[1], Farhana Hasan [3], Slawomir Lomnicki [3], Dorin Boldor [2], Robert Cook[1], Phillip Sprunger[4], Randall Hall[5], Stephania Cormier [6]

[1] Department of Chemistry, Louisiana State University, Baton Rouge, Louisiana 70803, USA

[2] Department of Engineering Science, Biological Engineering, Louisiana State University, Baton Rouge, Louisiana 70803, USA

[3] Department of Environmental Sciences, Louisiana State University, Baton Rouge, Louisiana 70803, USA

[4] Department of Physics and Astronomy, Louisiana State University, Baton Rouge, Louisiana 70803, USA

[5] Natural Sciences and Mathematics, School of Health and Natural Sciences, Dominican University of California, USA

[6] Department of Biological Sciences, LSU Superfund Research Program and Pennington Biomedical Research Center, Baton Rouge, Louisiana 70808, USA

The HHB (Higher Hydroxylated Batch) and **LHB** (Less Hydroxylated Batch) samples of 5%CuO/SiO_2 colored in green and gray, respectively.

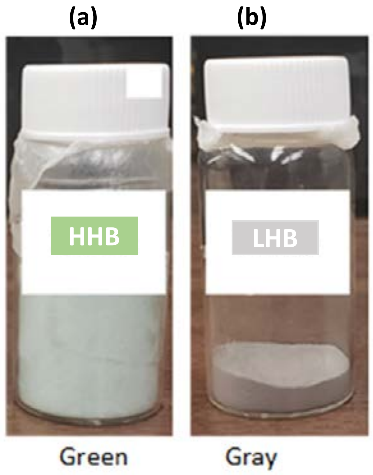

Figure S1. (a) HHB sample (extra water – 12mL, occasionally mixing and calcinated in air at 450 C for 6 hrs) and (b) LHB sample (extra water – 12mL, occasionally mixing and calcinated in air at 450 C for 4 days)

TEM images: Both HHB and LHB samples were scanned by TEM to investigate the presence of copper oxide nanoparticles supported on silica. **Figure S2 a, b** shows the presence of ultrafine nanoparticles that range in size from 5-8 nm supported on silica for **HHB**. The distribution of nanoparticles is not uniform on the substrate for the entire sample. In **Figures S2 c, d**, no nanoparticles were detected (**LHB sample**); this could be ascribed to particle growth and agglomeration through extended heating.

It was essential to investigate the oxidation states of copper supported on silica. Analogous data on the formation of Cu^{+1} following DCB exposure based on XRD measurements is presented in **Figures S3**.

XRD measurements: The XRD spectra shown in **Figure S3, (a)** (before exposure) and **S3, (b)** (after exposure) illustrate the effect of exposure to DCB on **HHB** sample.

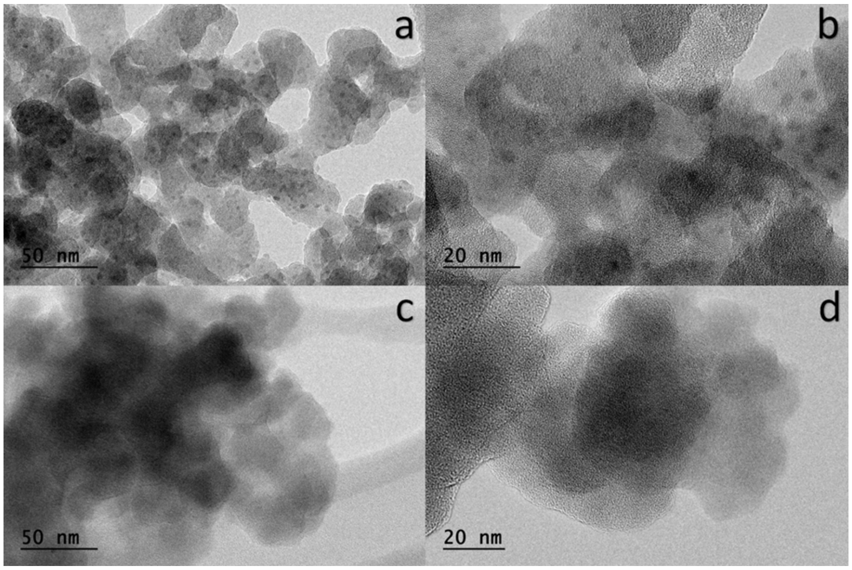

Figure S2. (a, b) - HHB exhibiting a **green** hue, displaying a distribution of nanosized CuO islands. (c, d) - LHB **gray** in color prepared in similar way as HHB but calcinated for 4 days; – the nanosized particles have not been detected.

At the beginning of the XRD spectra shown in **Figure S3, (a)** we can see a big hump at around 25°. This is ascribed to the amorphous silica support. The spectra following the hump starting 25° - 65° was carefully zoomed in to identify any peaks that could be attributed to the oxidation states of copper.

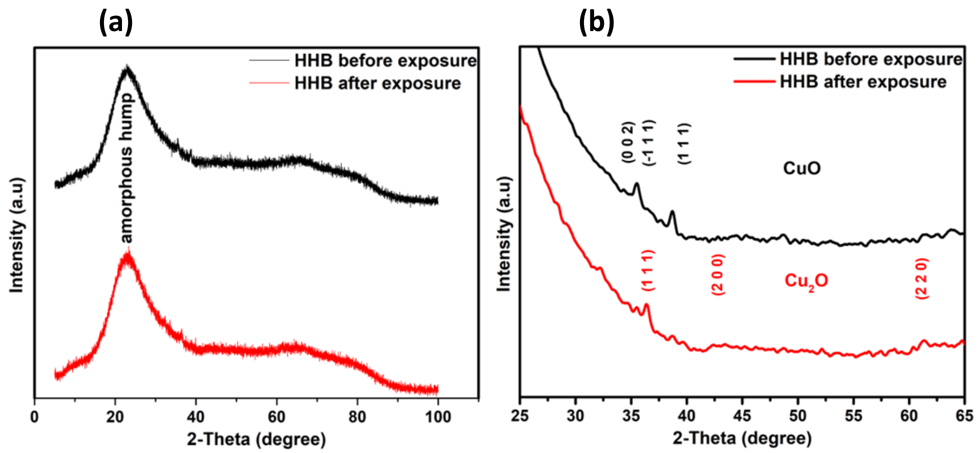

Figure S3: XRD spectra (a) full spectrum for HHB sample. (b) magnified spectrum after amorphous hump.

Due to the significant difference in ratio between silica and copper precursors used in synthesis, minor peaks at 35.4°, 35.5°, and 38.7° (black spectrum) were ascribed to CuO.

Upon exposure of the copper oxide/silica to DCB at 230 °C under vacuum weak peaks appearing at 36.4°, 42.3°, and 61.3° [1] characteristics for Cu^{+1} appeared, **Figure S3, (b)** red spectrum. It is important to highlight that numerous publications have successfully elucidated the reduction process of transition metals in the generation of EPFRs from various precursors using different techniques [2-8].

EPR spectra of DCB230 EPFRs

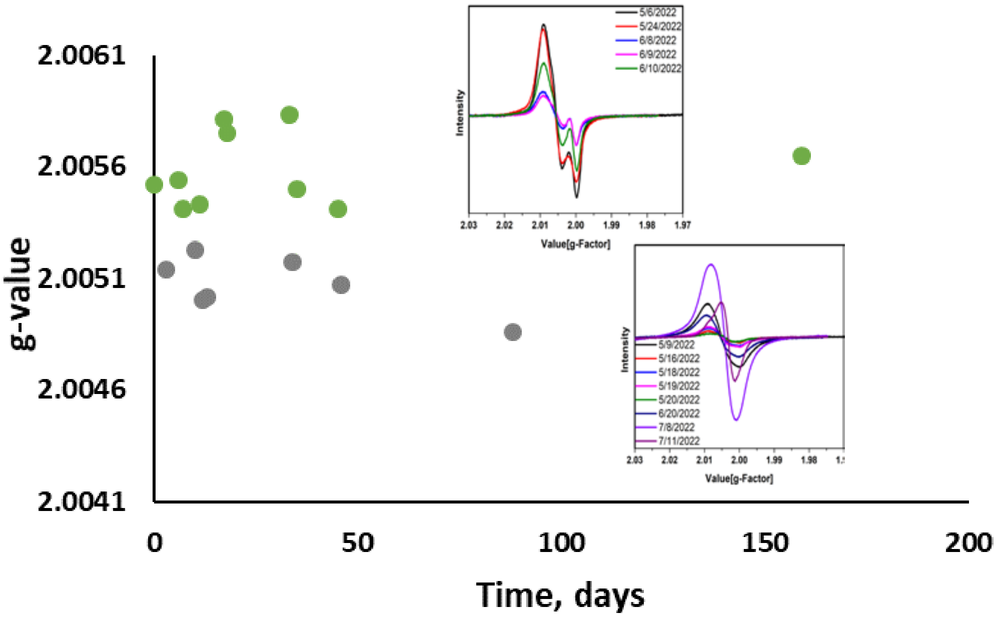

Figure S4. **Upper inset** - EPR spectra of DCB230 EPFRs with a split possessing higher than 2.0053(4) g values generated from exposure of the numerous HH-highly hydroxylated different samples (colored in green) during long period of measurements since 05.06.2022. **Below inset** - EPR spectra of DCB230 EPFRs without a split possessing less than 2.0053(4) g values generated from exposure of numerous LH-low hydroxylated different samples (colored in gray).

Isolated, monodentate (vicinal) and bidentate (geminal) adsorption. [9]

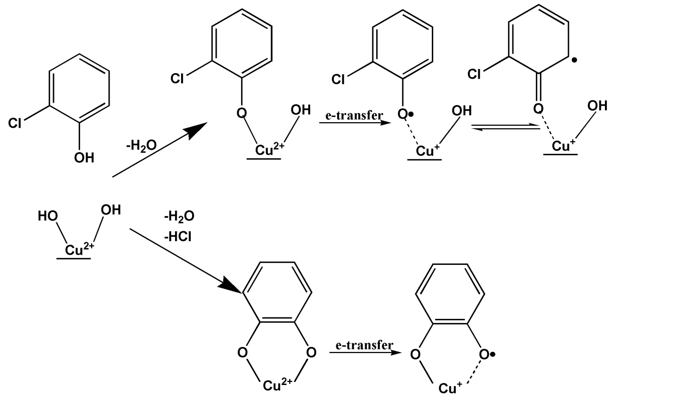

Figure S5. General Mechanism of EPFR formation from 2-monochlorophenol at Cu^{2+} sites on a CuO/silica particle.

EPR spectra of o-SQ radicals under dramatically different conditions[10].

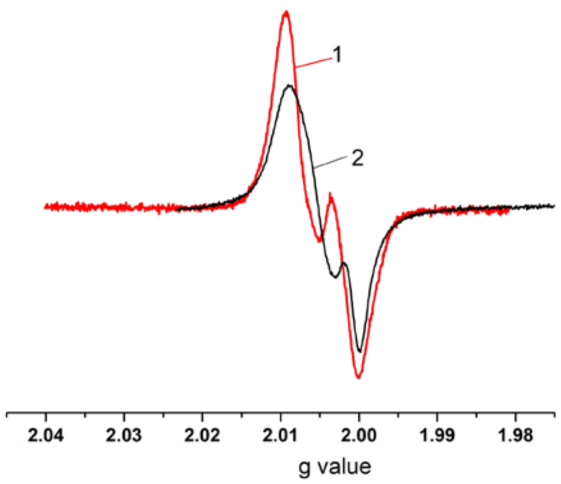

Figure S6. The red spectrum – EPR spectrum of the intermediate radicals detected cryogenically at liq. N2 temperature from vacuum pyrolysis of catechol at 650 oC[10]. The black spectrum – EPR spectrum of DCB230 EPFRs detected at room temperature using a catalyst 5%CuO/SiO_2 colored in green – this work.

Low yield DCB230 EPFRs overlaid on Cu^{+2} EPR spectrum, Fig. 7, (a). EasySpin Simulation, Fig.S 7, (b).[11]

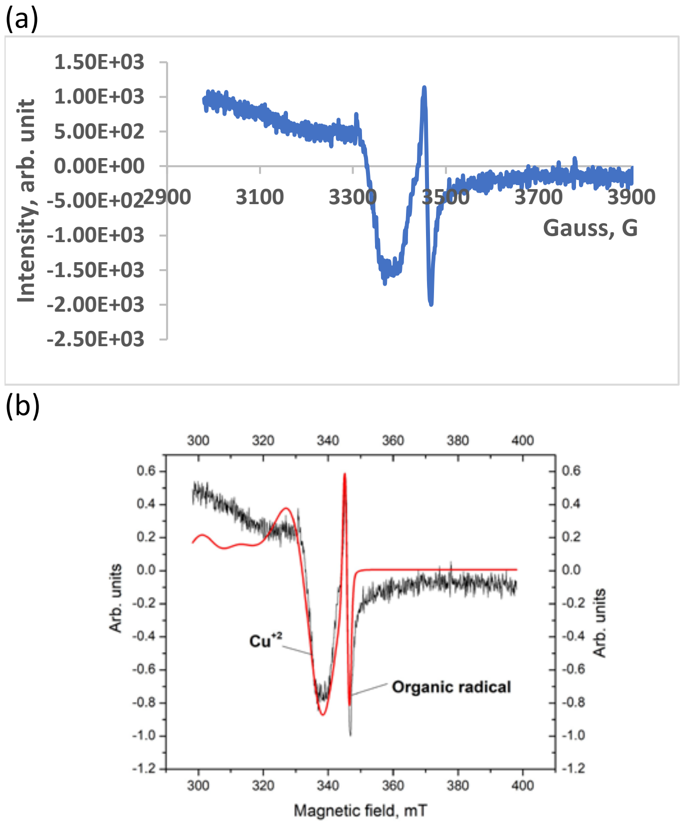

Fig. 7. (a) - DCB230 EPFRS EPR spectrum detected in magnetic field 2900-3900 G *. (b) - EasySpin simulation (red line) to fit experimental spectrum of DCB230 EPFRs (black line - organic radical overlaid on Cu^{+2} EPR spectrum) * generated in the laboratory. * 1 mt =10 gauss.

The EasySpin code (short version) is as following:

```
Cu.g = [2.0882 2.0882 2.390];
Cu.Nucs = 'Cu'; Cu.A = [52 461];% in Mhz;
Cu.lwpp = 7;% in mT; 1 mT = 10 G.
R.g = 2.0052;
R.lwpp = 1.5;%in mT;
Cu.weight = 0.9971;
R.weight = 0.0029;
Exp.mwFreq = 9.707; % in GHz;
Exp.Range = [298 398]; % in mT;
esfit(@pepper,spc,{Cu,R},{CuVary,VaryR},Exp);
```

Abbreviations in the code: g – EPR g values; A – hyperfine splitting in MHz for Cu; mT – militesla; R – organic radical; lwpp – EPR line width from peak to peak; esfit – EasySpin command for simulation; pepper –simulation for the solid state, spc – Bruker EPR file extension, % - for a note.

A reversible color change of the catalyst at exposure of DCB.

A noteworthy finding was the reversible color change of the catalyst, which was observed in various shades of green (see **Figure S8**, a, b, and d). After being exposed and pumped in the exposure chamber up to 10^{-2} torr for 30 minutes at room temperature, the catalyst turned almost white (as shown in Figure S8, c). However, during EPR measurements after 5 hours of aging, the green color slowly returned (with a stronger intensity than the initial catalyst, as depicted in Figure S8, d).

The color change of the catalyst (HHB) from different shades of the green color (**Figure S8)** can be attributed to a valence change of Cu^{+2}. The redox transformation of Cu^{+1}/Cu^{+2} in catalytic processes has been extensively studied and documented in numerous publications [12-13]. The fact that the DCB230 EPFRs sample also exhibits an intermediate white color (**Figure S8, c**) under vacuum further supports the formation of metastable Cu^{+1}, which rapidly converts to a light green color; according to the ref. [14], certain valence states of transition metals, such as monovalent Cu^{+1}, do not exhibit color. The return of green color occurs only when ambient air penetrates the

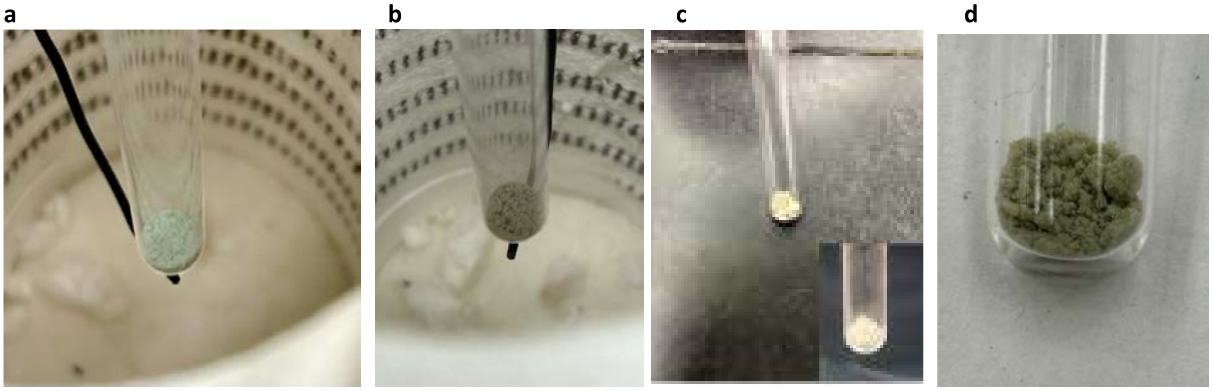

Figure S8. Color change of the catalyst 5%CuO/SiO_2; (a) - initial, (b) - after exposure by DCB at 230 °C, (c) – after exposure at room temperature, (d) – 5 hours aging when DCB230 EPFRs gone.

EPR tube containing DCB230 EPFRs. The oxygen oxidizes Cu^{+1}, which is formed during the chemisorption of DCB on the catalyst surfaces (Scheme 1, Figure 6 in the main text), reaction 1.

$$O_2 + Cu^{+1} \rightarrow O_2^{\bullet -} + Cu^{+2} \qquad (1)$$

The stronger green color of the catalyst after the complete annihilation of DCB230 EPFRs (Figure S8, d) supports the idea that Cu^{+2} complexes with more water molecules (as ligands are formed) compared to the initial ones, Figure S8, a.

On the other hand, the exact role of oxygen or water molecules on stability of EPFRs is not yet fully understood [15-16 17]. It is worth noting that the trace amount of water has a dual character in promoting or inhibiting the formation of EPFRs, as discussed in [18], which explains the formation of EPFRs on highly hydroxylated γ-Al_2O_3 (110) surfaces through a "water bridge" metastable mode.

Notably, the fact that bidentate chemisorption of DCB on the catalyst surface results in the release of a significant amount of HCl. Referring to ref[19], it has been demonstrated that HCl binds to the Cu^{+2}/Cu^{+1} cations in CuZSM-5 at low pressures. DFT calculations have suggested that the Cl atom binds to the mononuclear Cu+ site in the CuZSM-5 framework. Based on this scenario, it is possible that a similar process occurs with the catalyst discussed in this work, leading to the formation of a Cu(I)Cl salt, which is typically white in color.

Furthermore, it is also worth noting that the hydrogen cation from the adsorbed HCl may exhibit high mobility, allowing for the preferred reaction of adsorbed superoxide radicals to take place, reaction (2).

$$H^{+}_{ads} + O_2^{\cdot -}{}_{ads} \rightarrow HO_{2ads} \qquad (2)$$

Further secondary reactions of HO_{2ads} by formation of H_2O_2, possible chlorination reactions of adsorbed organic molecules[20] may occur as well; these secondary reactions are, however, not the focus of this current work.

References

1. Lee, S. Y.; Mettlach, N.; Nguyen, N.; Sun, Y. M.; White, J. M., Copper Oxide Reduction through Vacuum Annealing. *Applied Surface Science* **2003,** *206*, 102–109.
2. Farquar, G., Alderman, S., Poliakoff, E., Dellinger, B., X-Ray spectroscopic studies of the high-temperature reduction of Cu(II)O by 2-chlorophenol on a simulation fly-ash surface. *Environmenal Science and Technology* **2003**, 931-935.
3. Alderman, S. L., Farquar, G. R., Poliakoff, E. D., Dellinger, B., An infrared and X-ray spectroscopic study of the reactions of 2-chlorophenol, 1,2-dichlorobenzene, and chlorobenzene with model CuO/silica fly ash surfaces. *2005* **2005,** *39*, 7396-7401.
4. Lomnicki, S. M.; Wu, H. Y.; Osborne, S. N.; Pruett, J. M.; McCarley, R. L.; Poliakoff, E.; Dellinger, B., Size-selective synthesis of immobilized copper oxide nanoclusters on silica. *Materials Science and Engineering B-Advanced Functional Solid-State Materials* **2010,** *175* (2), 136-142.
5. Qin, L. J.; Yang, L. L.; Yang, J. H.; Weber, R.; Ranguelova, K.; Liu, X. Y.; Lin, B. C.; Li, C.; Zheng, M. H.; Liu, G. R., Photoinduced formation of persistent free radicals, hydrogen radicals, and hydroxyl radicals from catechol on atmospheric particulate matter. *Iscience* **2021,** *24* (3).
6. Yang, L. L.; Liu, G. R.; Zheng, M. H.; Jin, R.; Zhao, Y. Y.; Wu, X. L.; Xu, Y., Pivotal Roles of Metal Oxides in the Formation of Environmentally Persistent Free Radicals. *Environmental Science & Technology* **2017,** *51* (21), 12329-12336.
7. Jia, H. Z.; Zhao, S.; Shi, Y. F.; Zhu, L. Y.; Wang, C. Y.; Sharma, V. K., Transformation of Polycyclic Aromatic Hydrocarbons and Formation of Environmentally Persistent Free Radicals on Modified Montmorillonite: The Role of Surface Metal Ions and Polycyclic Aromatic Hydrocarbon Molecular Properties. *Environmental Science & Technology* **2018,** *52* (10), 5725-5733.
8. Liu, S. T.; Liu, G. R.; Yang, L. L.; Li, D.; Zheng, M. H., Critical influences of metal compounds on the formation and stabilization of environmentally persistent free radicals. *Chemical Engineering Journal* **2022,** *427*.
9. Kiruri, L. W.; khachatryan, L.; Dellinger, B.; Lomnicki, S., Effect of copper oxide concentration on the formation and persistency of environmentally persistent free radicals (EPFRs) in particulates. *Env.Sci.Technol.* **2014,** *48*, 2212-2217.
10. Khachatryan, L.; Adounkpe, J.; Asatryan R.; Dellinger, B., Radicals from the Gas-Phase Pyrolysis of Catechol: 1. o-Semiquinone and ipso-Catechol Radicals. *J.Phys. Chem., A* **2010,** *114* (6), 2306-2312.

11. Stoll, S.; Schweiger, A., EasySpin, a comprehensive software package for spectral simulation and analysis in EPR. *Journal of Magnetic Resonance* **2006,** *178* (1), 42-55.
12. Dooley, D. M.; Mcguirl, M. A.; Brown, D. E.; Turowski, P. N.; Mcintire, W. S.; Knowles, P. F., A Cu(I)-Semiquinone State in Substrate-Reduced Amine Oxidases. *Nature* **1991,** *349* (6306), 262-264.
13. Rajabimoghadam, K.; Darwish, Y.; Bashir, U.; Pitman, D.; Eichelberger, S.; Siegler, M. A.; Swart, M.; Garcia-Bosch, I., Catalytic Aerobic Oxidation of Alcohols by Copper Complexes Bearing Redox-Active Ligands with Tunable H-Bonding Groups. *Journal of the American Chemical Society* **2018,** *140* (48), 16625-16634.
14. Nassau, K., Origins of Color in Minerals. *American Mineralogist* **1978,** *63* (3-4), 219-229.
15. Maskos, Z.; Dellinger, B., Formation of the secondary radicals from the aging of tobacco smoke. *Energy & Fuels* **2008,** *22* (1), 382-388.
16. Feng, J.-W., Zheng, S., Maciel, G. E., EPR investigations of the effects of inorganic additives on the charring and char/air interactions of cellulose. *Energy & Fuels* **2004,** *18*, 1049-1065.
17. Pan, B.; Li, H.; Lang, D.; Xing, B. S., Environmentally persistent free radicals: Occurrence, formation mechanisms and implications. *Environmental Pollution* **2019,** *248*, 320-331.
18. Wei Wang; Ruiying Zhang; Zhenhua Liu; Wenxing Wang; Qingzhu Zhang; Qiao Wang, Periodic DFT calculation for the formation of EPFRs from phenol on γ-Al2O3 (110): Site-dependent mechanism and the role of ambient water. *Journal of Environmental Chemical Engineering* **2022,** *10* (5), 108386.
19. Mosallanejad, S.; Dlugogorski, B. Z.; Kennedy, E. M.; Stockenhuber, M., HCl Adsorption on Copper-Modified ZSM-5: FTIR and DFT Study. *Journal of Physical Chemistry C* **2013,** *117* (38), 19365-19372.
20. Vejerano, E. Formation and Stabilization of Combustion-Generated Environmentally Persistent Free Radicals on Transition Metal Oxides Supported on Silica. Dissertation. Louisiana State University, **2011**.
